# 3-(2,3,5,6,7,8-Hexahydro-1*H*-cyclo­penta­[*b*]quinolin-9-yl)-1,5-bis­(4-methoxy­phen­yl)biuret

**DOI:** 10.1107/S1600536810006057

**Published:** 2010-02-20

**Authors:** Kaori Sakurai, Keiichi Noguchi, Koichiro Nishibe

**Affiliations:** a Division of Biotechnology and Life Science, Institute of Symbiotic Science and Technology, Tokyo University of Agriculture and Technology, 2-24-16 Naka-cho, Koganei, Tokyo 184-8588, Japan

## Abstract

Ipidacrine (2,3,5,6,7,8-hexa­hydro-1*H*-cyclo­penta­[*b*]quinolin-9-amine) was reacted with 4-methoxy­phenyl isocyanate to give the title compound, C_28_H_30_N_4_O_4_. An intra­molecular N—H⋯O hydrogen bond results in an essentially planar [r.m.s. deviation from the mean plane is 0.126 (1) Å] conformation for the biuret unit. The central ring of the quinoline unit is twisted by 78.2 (1)° with respect to the biuret mean plane, whereas the two 4-methoxy­benzene rings are twisted out of this plane by 24.3 (1)° and 48.5 (1)°, resulting in an overall propeller-like structure. An inter­molecular N—H⋯N hydrogen bond between the biuret NH atom and the quinoline ring nitro­gen defines the crystal packing.

## Related literature

For related structures, see: Roh & Jeong (2000 ▶); Harrison (2007 ▶).
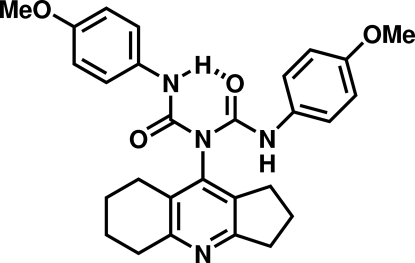


## Experimental

### 

#### Crystal data

C_28_H_30_N_4_O_4_
*M* *_r_* = 486.56
Monoclinic, 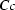

*a* = 22.4514 (4) Å
*b* = 12.7128 (2) Å
*c* = 8.83183 (16) Å
β = 105.526 (1)°
*V* = 2428.80 (8) Å^3^
*Z* = 4
Cu *K*α radiation
μ = 0.73 mm^−1^
*T* = 193 K
0.45 × 0.25 × 0.10 mm

#### Data collection

Rigaku R-AXIS RAPID diffractometer
Absorption correction: numerical (*ABSCOR*; Higashi, 1999 ▶) *T*_min_ = 0.754, *T*_max_ = 0.929
19342 measured reflections
2235 independent reflections
2168 reflections with *I* > 2σ(*I*)
*R*_int_ = 0.022

#### Refinement

*R*[*F* ^2^ > 2σ(*F* ^2^)] = 0.028
*wR*(*F* ^2^) = 0.072
*S* = 1.09
2235 reflections
328 parameters
2 restraints
H-atom parameters constrained
Δρ_max_ = 0.17 e Å^−3^
Δρ_min_ = −0.15 e Å^−3^

### 

Data collection: *PROCESS-AUTO* (Rigaku, 1998 ▶); cell refinement: *PROCESS-AUTO*; data reduction: *CrystalStructure* (Rigaku/MSC, 2004 ▶); program(s) used to solve structure: *SIR2004* (Burla *et al.*, 2005 ▶); program(s) used to refine structure: *SHELXL97* (Sheldrick, 2008 ▶); molecular graphics: *ORTEP-3 for Windows* (Farrugia, 1997 ▶); software used to prepare material for publication: *SHELXL97*.

## Supplementary Material

Crystal structure: contains datablocks I, global. DOI: 10.1107/S1600536810006057/fj2281sup1.cif

Structure factors: contains datablocks I. DOI: 10.1107/S1600536810006057/fj2281Isup2.hkl

Additional supplementary materials:  crystallographic information; 3D view; checkCIF report

## Figures and Tables

**Table 1:** Hydrogen-bond geometry (Å, °)

*D*—H⋯*A*	*D*—H	H⋯*A*	*D*⋯*A*	*D*—H⋯*A*
N2—H2⋯O3	0.88	1.97	2.623 (3)	130
N4—H4⋯N3^i^	0.88	2.26	2.961 (2)	137

Symmetry code: (i) 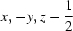.

**Fig. 1.**
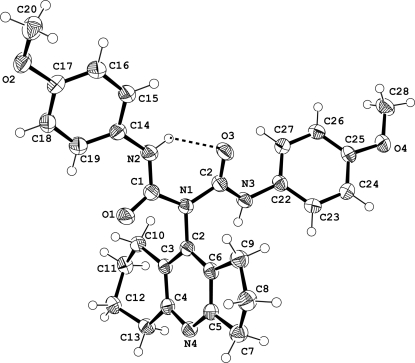
The molecular structure of (I). The ellipsoids are drawn at the 50% probability level and H atoms are shown as small spheres with arbitrary radii. The hydrogen bond is indicated by a dashed line.

## supplementary crystallographic information

### Comment

The title compound (I) (Fig. 1) was obtained as a side product in the synthesis of ipidacrine urea derivatives.

In the biuret moiety of (I), there are two types of C—N bonds, both of which display a partial double bond character. Their bond lengths are between 1.47 Å (for typical C—N bonds) and 1.28 Å (for C═N bonds) (Allen *et al.* 1987). The terminal C1—N2 and C21—N4 bonds are shorter (1.340 (3) and 1.343 (3) Å) than the internal C1—N1 and C21–N1 bonds (1.429 (2) and 1.415 (3) Å). O3 is involved in the hydrogen bonding with H2, forming a six-membered ring, which is consistent with solid state structures of other biuret compounds (Harrison, 2007; Roh and Jeong, 2000). However, the biuret moiety of (I) is not completely planar as the dihedral angle for O3–C21–N1–C1 is 8.8 (3)°.

Due to the partial double bond character of the terminal biuret C—N bonds, A^1,3^ strain is incurred between the buiret carbonyl groups and the bulky *p*-methoxyphenyl rings. These interactions cause the *p*-methoxyphenyl rings to twist out of plane with respect to the biuret moiety by approximately 24.3 (1)° and 48.5 (1)°. The bulkier quinoline moiety is substituted at N1, which forms a partial double bond with both C1 and C2. It develops A^1,3^ strain with two groups, one with *p*-methoxyphenylamino group, the other with one of the biuret carbonyl group. As a consequence, it is twisted close to perpendicular (78.2 (1)°) to the buiret plane. The steric congestion among the three aromatic substituents around the biuret moiety drives (I) to adopt an overall propeller-like structure.

In the present crystal structure for the title compound (I), these two *p*-methoxyphenyl rings are not geometrically equivalent. However, the ^1^H NMR spectrum of (I) shows only one set of peaks for the protons of a *p*-methoxyphenyl group. This observation suggests that the hydrogen bonds for O1···H4 and O3···H2 are in fast exchange in solution and that the rotational barrier around the internal C—N bond of the biuret group is not significant under ambient condition. Since the biuret moiety deviates slightly from a planar conformation, there is helicity along the biuret backbone (N2—C1—N2—C21—N4). The interconversion of the two hydrogen bonding pairs (between O1···H4 and O3···H2) represents the interconversion of two corresponding helical conformations of (I), making the molecule dynamically racemic in solution.

### Experimental

The title compound was prepared by reacting ipidacrine (20.0 mg, 0.11 mmol) and 4-methoxyphenyl isocyanate (23.9 mg, 0.16 mmol) in dichloromethane (0.5 ml) at room temperature for 18 h. The resultant reaction mixture was concentrated in vacuo and was purified by flash chromatography (2 % MeOH/CH_2_Cl_2_) to afford the title compound I (18.1 mg; 33.8 % yield). Crystals suitable for X-ray diffraction were obtained by slow evaporation of the solution of (I) in MeCN. ^1^H NMR (CDCl_3_) δ 7.28 (d, J = 8.96 Hz, 4H), δ (d, *J* = 8.96 Hz, 4H), δ 3.82 (s, 1H), δ 3.10 (dd, *J* = 7.54 Hz, 2H), δ 2.94-3.00 (m, 4H), δ 2.71 (m, 2H), δ 2.18 (m, 2H), δ 1.87 (m, 4H). ESI-MS calcd for C_28_H_31_N_4_O_4_ (M+H^+^) 487.23, found 487.22.

### Refinement

The value of the absolute structure parameter is meaningless because of its large s.u. value (Flack's x = -0.01 (14)). Therefore, the merging of Friedel pair data was performed before the final refinement cycles. The methylene, methyl and phenyl H atoms were positioned using the HFIX 23, HFIX 137 and HFIX 43 instructions, with C—H = 0.99, 0.98 and 0.95 Å, respectively. In addition, the amide H atoms were positioned using the HFIX 43 instructions, with N—H = 0.88 Å. These C- and N-bound H atoms were also refined as a riding model, with *U*_iso_(H) = 1.2*U*_eq_(C or N).

### Crystal data

**Table:**

C_28_H_30_N_4_O_4_	*F*(000) = 1032
*M**_r_* = 486.56	*D*_x_ = 1.331 Mg m^−^^3^
Monoclinic, *C**c*	Cu *K*α radiation, λ = 1.54187 Å
Hall symbol: C -2yc	Cell parameters from 18332 reflections
*a* = 22.4514 (4) Å	θ = 4.0–68.2°
*b* = 12.7128 (2) Å	µ = 0.73 mm^−^^1^
*c* = 8.83183 (16) Å	*T* = 193 K
β = 105.526 (1)°	Block, colorless
*V* = 2428.80 (8) Å^3^	0.45 × 0.25 × 0.10 mm
*Z* = 4	

### Data collection

**Table:**

Rigaku R-AXIS RAPID diffractometer	2235 independent reflections
Radiation source: rotating anode	2168 reflections with *I* > 2σ(*I*)
graphite	*R*_int_ = 0.022
Detector resolution: 10.00 pixels mm^-1^	θ_max_ = 68.2°, θ_min_ = 4.0°
ω scans	*h* = −26→26
Absorption correction: numerical (*ABSCOR*; Higashi, 1999)	*k* = −15→15
*T*_min_ = 0.754, *T*_max_ = 0.929	*l* = −10→10
19342 measured reflections	

### Refinement

**Table:**

Refinement on *F*^2^	Secondary atom site location: difference Fourier map
Least-squares matrix: full	Hydrogen site location: inferred from neighbouring sites
*R*[*F*^2^ > 2σ(*F*^2^)] = 0.028	H-atom parameters constrained
*wR*(*F*^2^) = 0.072	*w* = 1/[σ^2^(*F*_o_^2^) + (0.0431*P*)^2^ + 0.5591*P*] where *P* = (*F*_o_^2^ + 2*F*_c_^2^)/3
*S* = 1.09	(Δ/σ)_max_ < 0.001
2235 reflections	Δρ_max_ = 0.17 e Å^−^^3^
328 parameters	Δρ_min_ = −0.15 e Å^−^^3^
2 restraints	Extinction correction: *SHELXL*, Fc^*^=kFc[1+0.001xFc^2^λ^3^/sin(2θ)]^-1/4^
Primary atom site location: structure-invariant direct methods	Extinction coefficient: 0.00173 (14)

### Special details

**Table:**

Geometry. All esds (except the esd in the dihedral angle between two l.s. planes) are estimated using the full covariance matrix. The cell esds are taken into account individually in the estimation of esds in distances, angles and torsion angles; correlations between esds in cell parameters are only used when they are defined by crystal symmetry. An approximate (isotropic) treatment of cell esds is used for estimating esds involving l.s. planes.
Refinement. Refinement of *F*^2^ against ALL reflections. The weighted *R*-factor wR and goodness of fit *S* are based on *F*^2^, conventional *R*-factors *R* are based on *F*, with *F* set to zero for negative *F*^2^. The threshold expression of *F*^2^ > σ(*F*^2^) is used only for calculating *R*-factors(gt) etc. and is not relevant to the choice of reflections for refinement. *R*-factors based on *F*^2^ are statistically about twice as large as those based on *F*, and *R*- factors based on ALL data will be even larger.

### Fractional atomic coordinates and isotropic or equivalent isotropic displacement parameters (Å^2^)

**Table:**

	*x*	*y*	*z*	*U*_iso_*/*U*_eq_	
O1	0.06234 (8)	0.24673 (12)	1.0794 (2)	0.0445 (4)	
O2	−0.10168 (7)	0.65103 (12)	1.1352 (2)	0.0444 (4)	
O3	0.17237 (8)	0.40771 (11)	0.8629 (2)	0.0430 (4)	
O4	0.38341 (7)	0.39432 (13)	0.50693 (18)	0.0416 (4)	
N1	0.13344 (8)	0.25410 (12)	0.9380 (2)	0.0306 (4)	
N2	0.06980 (9)	0.39868 (14)	0.9509 (2)	0.0395 (4)	
H2	0.0880	0.4276	0.8845	0.047*	
N3	0.18591 (8)	−0.05082 (13)	1.12185 (19)	0.0305 (4)	
N4	0.20716 (8)	0.25153 (13)	0.7982 (2)	0.0329 (4)	
H4	0.2030	0.1827	0.7998	0.039*	
C1	0.08617 (9)	0.30000 (16)	0.9974 (2)	0.0321 (4)	
C2	0.15121 (9)	0.14855 (15)	0.9958 (2)	0.0284 (4)	
C3	0.11494 (9)	0.06101 (15)	0.9357 (2)	0.0281 (4)	
C4	0.13470 (9)	−0.03777 (15)	1.0012 (2)	0.0292 (4)	
C5	0.21934 (10)	0.03451 (15)	1.1761 (2)	0.0309 (4)	
C6	0.20443 (9)	0.13524 (16)	1.1171 (2)	0.0305 (4)	
C7	0.27760 (11)	0.03514 (17)	1.3082 (3)	0.0383 (5)	
H7A	0.3131	0.0083	1.2731	0.046*	
H7B	0.2729	−0.0077	1.3979	0.046*	
C8	0.28579 (11)	0.15237 (18)	1.3523 (3)	0.0426 (5)	
H8A	0.3302	0.1715	1.3835	0.051*	
H8B	0.2679	0.1683	1.4408	0.051*	
C9	0.25137 (11)	0.21355 (17)	1.2040 (3)	0.0393 (5)	
H9A	0.2311	0.2772	1.2316	0.047*	
H9B	0.2798	0.2345	1.1410	0.047*	
C10	0.05380 (10)	0.07332 (17)	0.8131 (2)	0.0340 (5)	
H10A	0.0591	0.1235	0.7320	0.041*	
H10B	0.0234	0.1040	0.8635	0.041*	
C11	0.02802 (11)	−0.02892 (18)	0.7335 (3)	0.0367 (5)	
H11A	0.0514	−0.0494	0.6577	0.044*	
H11B	−0.0157	−0.0186	0.6743	0.044*	
C12	0.03227 (10)	−0.11677 (17)	0.8540 (3)	0.0353 (5)	
H12A	0.0093	−0.0964	0.9307	0.042*	
H12B	0.0135	−0.1819	0.8004	0.042*	
C13	0.09972 (10)	−0.13645 (16)	0.9390 (3)	0.0343 (5)	
H13A	0.1018	−0.1852	1.0277	0.041*	
H13B	0.1201	−0.1712	0.8658	0.041*	
C14	0.02570 (10)	0.46052 (17)	0.9997 (3)	0.0348 (5)	
C15	0.03188 (11)	0.56866 (18)	0.9956 (3)	0.0429 (5)	
H15	0.0651	0.5981	0.9619	0.051*	
C16	−0.00968 (11)	0.63476 (18)	1.0399 (3)	0.0432 (5)	
H16	−0.0048	0.7089	1.0370	0.052*	
C17	−0.05833 (10)	0.59240 (17)	1.0885 (3)	0.0359 (5)	
C18	−0.06523 (10)	0.48428 (18)	1.0894 (3)	0.0414 (5)	
H18	−0.0990	0.4549	1.1208	0.050*	
C19	−0.02389 (11)	0.41839 (19)	1.0454 (3)	0.0429 (5)	
H19	−0.0293	0.3443	1.0464	0.051*	
C20	−0.08528 (13)	0.7562 (2)	1.1797 (4)	0.0531 (6)	
H20A	−0.1159	0.7867	1.2277	0.064*	
H20B	−0.0840	0.7972	1.0866	0.064*	
H20C	−0.0445	0.7575	1.2556	0.064*	
C21	0.17192 (10)	0.31169 (16)	0.8647 (3)	0.0324 (4)	
C22	0.25105 (9)	0.29474 (15)	0.7251 (2)	0.0308 (4)	
C23	0.31085 (10)	0.25400 (15)	0.7664 (2)	0.0326 (4)	
H23	0.3220	0.2016	0.8456	0.039*	
C24	0.35403 (10)	0.28971 (17)	0.6923 (2)	0.0340 (4)	
H24	0.3948	0.2619	0.7207	0.041*	
C25	0.33780 (10)	0.36646 (16)	0.5759 (2)	0.0326 (4)	
C26	0.27881 (10)	0.40864 (16)	0.5364 (2)	0.0347 (5)	
H26	0.2679	0.4622	0.4589	0.042*	
C27	0.23551 (10)	0.37212 (17)	0.6111 (3)	0.0343 (4)	
H27	0.1949	0.4006	0.5836	0.041*	
C28	0.36885 (12)	0.4734 (2)	0.3897 (3)	0.0483 (6)	
H28A	0.4050	0.4872	0.3506	0.058*	
H28B	0.3570	0.5380	0.4346	0.058*	
H28C	0.3344	0.4496	0.3027	0.058*	

### Atomic displacement parameters (Å^2^)

**Table:**

	*U*^11^	*U*^22^	*U*^33^	*U*^12^	*U*^13^	*U*^23^
O1	0.0526 (10)	0.0364 (8)	0.0552 (10)	0.0085 (7)	0.0329 (8)	0.0106 (7)
O2	0.0345 (8)	0.0379 (8)	0.0634 (10)	0.0020 (6)	0.0175 (7)	−0.0103 (7)
O3	0.0495 (9)	0.0270 (7)	0.0621 (10)	−0.0047 (7)	0.0315 (8)	−0.0014 (7)
O4	0.0372 (8)	0.0457 (9)	0.0458 (9)	−0.0037 (7)	0.0177 (7)	0.0070 (7)
N1	0.0318 (8)	0.0258 (8)	0.0365 (9)	0.0002 (7)	0.0133 (7)	0.0008 (7)
N2	0.0456 (10)	0.0323 (9)	0.0489 (11)	0.0058 (8)	0.0267 (9)	0.0082 (8)
N3	0.0330 (9)	0.0274 (8)	0.0326 (9)	0.0005 (7)	0.0115 (7)	−0.0006 (7)
N4	0.0376 (10)	0.0258 (8)	0.0387 (9)	−0.0028 (7)	0.0161 (8)	−0.0007 (7)
C1	0.0343 (11)	0.0299 (10)	0.0341 (10)	0.0005 (8)	0.0126 (9)	−0.0007 (8)
C2	0.0308 (10)	0.0264 (9)	0.0313 (10)	0.0009 (8)	0.0142 (8)	−0.0012 (7)
C3	0.0287 (10)	0.0298 (9)	0.0277 (9)	−0.0012 (8)	0.0111 (8)	−0.0005 (8)
C4	0.0287 (10)	0.0320 (10)	0.0290 (10)	−0.0012 (8)	0.0116 (8)	−0.0024 (8)
C5	0.0299 (10)	0.0329 (10)	0.0321 (10)	0.0016 (8)	0.0122 (8)	0.0004 (8)
C6	0.0313 (10)	0.0300 (10)	0.0324 (10)	−0.0028 (8)	0.0124 (8)	−0.0030 (8)
C7	0.0365 (11)	0.0382 (11)	0.0380 (11)	−0.0001 (9)	0.0059 (10)	−0.0001 (9)
C8	0.0379 (12)	0.0426 (12)	0.0432 (13)	−0.0052 (10)	0.0040 (10)	−0.0052 (10)
C9	0.0388 (12)	0.0338 (11)	0.0428 (12)	−0.0059 (9)	0.0066 (10)	−0.0025 (9)
C10	0.0321 (11)	0.0364 (11)	0.0329 (10)	0.0004 (9)	0.0075 (9)	0.0035 (9)
C11	0.0360 (11)	0.0426 (12)	0.0316 (11)	−0.0064 (9)	0.0092 (9)	−0.0026 (9)
C12	0.0366 (11)	0.0365 (11)	0.0341 (11)	−0.0075 (9)	0.0114 (9)	−0.0054 (9)
C13	0.0397 (12)	0.0285 (10)	0.0358 (11)	−0.0022 (9)	0.0118 (9)	−0.0029 (8)
C14	0.0353 (11)	0.0341 (11)	0.0366 (11)	0.0053 (9)	0.0126 (9)	0.0033 (9)
C15	0.0441 (13)	0.0343 (12)	0.0579 (14)	0.0029 (10)	0.0268 (11)	0.0065 (10)
C16	0.0444 (13)	0.0303 (11)	0.0587 (15)	0.0021 (10)	0.0205 (11)	0.0021 (10)
C17	0.0319 (11)	0.0376 (11)	0.0374 (11)	0.0054 (9)	0.0080 (9)	−0.0022 (9)
C18	0.0352 (12)	0.0392 (12)	0.0543 (13)	−0.0015 (9)	0.0200 (10)	−0.0020 (10)
C19	0.0428 (13)	0.0322 (11)	0.0574 (15)	0.0007 (9)	0.0198 (11)	0.0009 (10)
C20	0.0440 (13)	0.0434 (13)	0.0753 (18)	−0.0013 (11)	0.0218 (13)	−0.0171 (12)
C21	0.0328 (10)	0.0308 (10)	0.0352 (10)	−0.0028 (8)	0.0120 (8)	−0.0007 (8)
C22	0.0337 (10)	0.0285 (10)	0.0326 (10)	−0.0036 (8)	0.0131 (8)	−0.0034 (8)
C23	0.0383 (12)	0.0284 (10)	0.0313 (10)	0.0006 (8)	0.0097 (9)	0.0027 (8)
C24	0.0293 (10)	0.0362 (11)	0.0358 (10)	0.0022 (9)	0.0076 (8)	−0.0006 (9)
C25	0.0331 (10)	0.0331 (10)	0.0323 (10)	−0.0073 (8)	0.0103 (8)	−0.0038 (8)
C26	0.0397 (12)	0.0312 (10)	0.0338 (11)	0.0001 (9)	0.0113 (9)	0.0045 (8)
C27	0.0311 (10)	0.0333 (10)	0.0386 (11)	0.0008 (8)	0.0095 (9)	0.0011 (9)
C28	0.0562 (15)	0.0430 (13)	0.0532 (14)	−0.0043 (11)	0.0275 (12)	0.0090 (11)

### Geometric parameters (Å, °)

**Table:**

O1—C1	1.215 (3)	C10—H10B	0.9900
O2—C17	1.374 (3)	C11—C12	1.528 (3)
O2—C20	1.414 (3)	C11—H11A	0.9900
O3—C21	1.221 (3)	C11—H11B	0.9900
O4—C25	1.371 (3)	C12—C13	1.520 (3)
O4—C28	1.417 (3)	C12—H12A	0.9900
N1—C21	1.415 (3)	C12—H12B	0.9900
N1—C1	1.429 (2)	C13—H13A	0.9900
N1—C2	1.453 (2)	C13—H13B	0.9900
N2—C1	1.340 (3)	C14—C15	1.383 (3)
N2—C14	1.418 (3)	C14—C19	1.389 (3)
N2—H2	0.8800	C15—C16	1.388 (3)
N3—C5	1.333 (3)	C15—H15	0.9500
N3—C4	1.352 (3)	C16—C17	1.385 (3)
N4—C21	1.343 (3)	C16—H16	0.9500
N4—C22	1.425 (3)	C17—C18	1.383 (3)
N4—H4	0.8800	C18—C19	1.381 (3)
C2—C6	1.385 (3)	C18—H18	0.9500
C2—C3	1.397 (3)	C19—H19	0.9500
C3—C4	1.404 (3)	C20—H20A	0.9800
C3—C10	1.512 (3)	C20—H20B	0.9800
C4—C13	1.504 (3)	C20—H20C	0.9800
C5—C6	1.390 (3)	C22—C27	1.383 (3)
C5—C7	1.502 (3)	C22—C23	1.393 (3)
C6—C9	1.503 (3)	C23—C24	1.383 (3)
C7—C8	1.539 (3)	C23—H23	0.9500
C7—H7A	0.9900	C24—C25	1.393 (3)
C7—H7B	0.9900	C24—H24	0.9500
C8—C9	1.542 (3)	C25—C26	1.384 (3)
C8—H8A	0.9900	C26—C27	1.393 (3)
C8—H8B	0.9900	C26—H26	0.9500
C9—H9A	0.9900	C27—H27	0.9500
C9—H9B	0.9900	C28—H28A	0.9800
C10—C11	1.517 (3)	C28—H28B	0.9800
C10—H10A	0.9900	C28—H28C	0.9800
			
C17—O2—C20	116.32 (17)	C11—C12—H12A	109.8
C25—O4—C28	117.02 (17)	C13—C12—H12B	109.8
C21—N1—C1	124.17 (16)	C11—C12—H12B	109.8
C21—N1—C2	119.67 (16)	H12A—C12—H12B	108.2
C1—N1—C2	114.15 (16)	C4—C13—C12	113.39 (17)
C1—N2—C14	125.59 (18)	C4—C13—H13A	108.9
C1—N2—H2	117.2	C12—C13—H13A	108.9
C14—N2—H2	117.2	C4—C13—H13B	108.9
C5—N3—C4	117.49 (17)	C12—C13—H13B	108.9
C21—N4—C22	122.56 (16)	H13A—C13—H13B	107.7
C21—N4—H4	118.7	C15—C14—C19	119.0 (2)
C22—N4—H4	118.7	C15—C14—N2	117.37 (19)
O1—C1—N2	125.17 (19)	C19—C14—N2	123.6 (2)
O1—C1—N1	118.70 (18)	C14—C15—C16	121.0 (2)
N2—C1—N1	116.06 (17)	C14—C15—H15	119.5
C6—C2—C3	119.41 (18)	C16—C15—H15	119.5
C6—C2—N1	118.90 (17)	C17—C16—C15	119.8 (2)
C3—C2—N1	121.66 (18)	C17—C16—H16	120.1
C2—C3—C4	117.90 (18)	C15—C16—H16	120.1
C2—C3—C10	120.99 (18)	O2—C17—C18	116.60 (19)
C4—C3—C10	120.93 (17)	O2—C17—C16	124.26 (19)
N3—C4—C3	122.87 (17)	C18—C17—C16	119.1 (2)
N3—C4—C13	115.87 (17)	C19—C18—C17	121.1 (2)
C3—C4—C13	121.26 (18)	C19—C18—H18	119.5
N3—C5—C6	123.98 (19)	C17—C18—H18	119.5
N3—C5—C7	124.98 (18)	C18—C19—C14	119.9 (2)
C6—C5—C7	111.04 (18)	C18—C19—H19	120.0
C2—C6—C5	118.31 (18)	C14—C19—H19	120.0
C2—C6—C9	131.00 (19)	O2—C20—H20A	109.5
C5—C6—C9	110.68 (19)	O2—C20—H20B	109.5
C5—C7—C8	102.83 (18)	H20A—C20—H20B	109.5
C5—C7—H7A	111.2	O2—C20—H20C	109.5
C8—C7—H7A	111.2	H20A—C20—H20C	109.5
C5—C7—H7B	111.2	H20B—C20—H20C	109.5
C8—C7—H7B	111.2	O3—C21—N4	123.77 (19)
H7A—C7—H7B	109.1	O3—C21—N1	122.10 (18)
C7—C8—C9	105.95 (18)	N4—C21—N1	114.13 (17)
C7—C8—H8A	110.5	C27—C22—C23	119.49 (19)
C9—C8—H8A	110.5	C27—C22—N4	122.23 (19)
C7—C8—H8B	110.5	C23—C22—N4	118.22 (18)
C9—C8—H8B	110.5	C24—C23—C22	120.15 (19)
H8A—C8—H8B	108.7	C24—C23—H23	119.9
C6—C9—C8	102.91 (17)	C22—C23—H23	119.9
C6—C9—H9A	111.2	C23—C24—C25	120.05 (19)
C8—C9—H9A	111.2	C23—C24—H24	120.0
C6—C9—H9B	111.2	C25—C24—H24	120.0
C8—C9—H9B	111.2	O4—C25—C26	124.47 (19)
H9A—C9—H9B	109.1	O4—C25—C24	115.45 (18)
C3—C10—C11	113.83 (18)	C26—C25—C24	120.08 (19)
C3—C10—H10A	108.8	C25—C26—C27	119.57 (19)
C11—C10—H10A	108.8	C25—C26—H26	120.2
C3—C10—H10B	108.8	C27—C26—H26	120.2
C11—C10—H10B	108.8	C22—C27—C26	120.6 (2)
H10A—C10—H10B	107.7	C22—C27—H27	119.7
C10—C11—C12	110.99 (18)	C26—C27—H27	119.7
C10—C11—H11A	109.4	O4—C28—H28A	109.5
C12—C11—H11A	109.4	O4—C28—H28B	109.5
C10—C11—H11B	109.4	H28A—C28—H28B	109.5
C12—C11—H11B	109.4	O4—C28—H28C	109.5
H11A—C11—H11B	108.0	H28A—C28—H28C	109.5
C13—C12—C11	109.42 (18)	H28B—C28—H28C	109.5
C13—C12—H12A	109.8		
			
C14—N2—C1—O1	5.5 (4)	C10—C11—C12—C13	−62.0 (2)
C14—N2—C1—N1	−177.6 (2)	N3—C4—C13—C12	157.07 (17)
C21—N1—C1—O1	−168.7 (2)	C3—C4—C13—C12	−23.4 (3)
C2—N1—C1—O1	−5.0 (3)	C11—C12—C13—C4	49.9 (2)
C21—N1—C1—N2	14.2 (3)	C1—N2—C14—C15	154.1 (2)
C2—N1—C1—N2	177.95 (18)	C1—N2—C14—C19	−28.2 (4)
C21—N1—C2—C6	64.4 (2)	C19—C14—C15—C16	1.6 (4)
C1—N1—C2—C6	−100.1 (2)	N2—C14—C15—C16	179.4 (2)
C21—N1—C2—C3	−117.7 (2)	C14—C15—C16—C17	−0.3 (4)
C1—N1—C2—C3	77.8 (2)	C20—O2—C17—C18	161.6 (2)
C6—C2—C3—C4	−0.4 (3)	C20—O2—C17—C16	−19.2 (3)
N1—C2—C3—C4	−178.32 (17)	C15—C16—C17—O2	179.7 (2)
C6—C2—C3—C10	174.75 (17)	C15—C16—C17—C18	−1.0 (3)
N1—C2—C3—C10	−3.1 (3)	O2—C17—C18—C19	−179.6 (2)
C5—N3—C4—C3	−1.9 (3)	C16—C17—C18—C19	1.1 (4)
C5—N3—C4—C13	177.58 (18)	C17—C18—C19—C14	0.2 (4)
C2—C3—C4—N3	1.9 (3)	C15—C14—C19—C18	−1.5 (4)
C10—C3—C4—N3	−173.28 (17)	N2—C14—C19—C18	−179.2 (2)
C2—C3—C4—C13	−177.55 (18)	C22—N4—C21—O3	3.1 (3)
C10—C3—C4—C13	7.3 (3)	C22—N4—C21—N1	−176.96 (18)
C4—N3—C5—C6	0.5 (3)	C1—N1—C21—O3	8.8 (3)
C4—N3—C5—C7	−179.63 (19)	C2—N1—C21—O3	−154.1 (2)
C3—C2—C6—C5	−0.9 (3)	C1—N1—C21—N4	−171.12 (18)
N1—C2—C6—C5	177.05 (16)	C2—N1—C21—N4	26.0 (3)
C3—C2—C6—C9	−179.65 (19)	C21—N4—C22—C27	−52.2 (3)
N1—C2—C6—C9	−1.7 (3)	C21—N4—C22—C23	130.4 (2)
N3—C5—C6—C2	0.9 (3)	C27—C22—C23—C24	−0.9 (3)
C7—C5—C6—C2	−179.00 (18)	N4—C22—C23—C24	176.55 (18)
N3—C5—C6—C9	179.93 (19)	C22—C23—C24—C25	−0.1 (3)
C7—C5—C6—C9	0.0 (2)	C28—O4—C25—C26	1.4 (3)
N3—C5—C7—C8	−164.38 (19)	C28—O4—C25—C24	−179.1 (2)
C6—C5—C7—C8	15.5 (2)	C23—C24—C25—O4	−178.30 (18)
C5—C7—C8—C9	−24.5 (2)	C23—C24—C25—C26	1.3 (3)
C2—C6—C9—C8	163.3 (2)	O4—C25—C26—C27	178.06 (19)
C5—C6—C9—C8	−15.5 (2)	C24—C25—C26—C27	−1.5 (3)
C7—C8—C9—C6	24.6 (2)	C23—C22—C27—C26	0.7 (3)
C2—C3—C10—C11	166.33 (18)	N4—C22—C27—C26	−176.64 (18)
C4—C3—C10—C11	−18.6 (3)	C25—C26—C27—C22	0.5 (3)
C3—C10—C11—C12	45.9 (2)		

### Hydrogen-bond geometry (Å, °)

**Table:**

*D*—H···*A*	*D*—H	H···*A*	*D*···*A*	*D*—H···*A*
N2—H2···O3	0.88	1.97	2.623 (3)	130
N4—H4···N3^i^	0.88	2.26	2.961 (2)	137

Symmetry codes: (i) *x*, −*y*, *z*−1/2.
